# Amyloid-β PET scans, economic strain and financial decision-making among persons with cognitive impairment and care partners: a mixed-methods analysis of the CARE-IDEAS study

**DOI:** 10.1186/s13195-026-02010-x

**Published:** 2026-04-14

**Authors:** Courtney H. Van Houtven, Cassie B. Ford, Elyse Couch, Megan Shepherd-Banigan, Wenhan Zhang, Emmanuelle Bélanger, Brenda L. Plassman, James R. Burke, Corinna Sorenson, Nicole DePasquale, Emily O’Brien, Eric Jutkowitz, Terrie Wetle, Valerie A. Smith

**Affiliations:** 1 Department of Population Health Sciences, Duke University, 215 Morris Street, Durham, NC 27701 USA; 2 Duke-Margolis Institute for Health Policy, Duke University, 100 Fuqua Drive, Durham, NC 27701 USA; 3 Center for Gerontology and Healthcare Research, Brown University School of Public Health, 121 South Main Street, 6th Fl, Providence, RI 02903 USA; 4 Department of Health Services, Policy and Practice, Brown University School of Public Health, 121 South Main Street, 6th Fl, Providence, RI 02903 USA; 5 Center of Innovation to Accelerate Discovery and Practice Transformation, Durham VA Health Care System, 508 Fulton St Mailcode 152, Durham, NC 27705 USA; 6 Department of Psychiatry and Behavioral Sciences, Duke University School of Medicine, Box 102508, Durham, NC 27710 USA; 7 Department of Neurology, Duke University School of Medicine, 200 Trent Dr Ste 1255, Durham, NC 27710 USA; 8 Duke Clinical Research Institute, School of Medicine, Duke University, Box 3850, Durham, NC 27710 USA; 9 Sanford School of Public Policy, Duke University, 201 Science Dr, Durham, NC 27708 USA; 10 Division of General Internal Medicine, Duke University School of Medicine, 710 W Main St, 1st Floor, Durham, NC 27701 USA; 11 Department of Biostatistics and Bioinformatics, Duke University School of Medicine, Durham, NC USA

**Keywords:** Amyloid-β, Alzheimer disease, Disclosure, Caregivers, Sspouses, Dementia, Economic strain, Mild cognitive impairment, Financial decision-making

## Abstract

**Background:**

To explore the relationship between receipt of amyloid-β PET scan results and subsequent experiences of economic strain and financial decision-making for persons with cognitive impairment and their care partners.

**Methods:**

A parallel convergent mixed-methods design where quantitative and qualitative data were simultaneously collected and analyzed. Participants included a subset of community-residing Medicare beneficiaries with cognitive impairment who had an amyloid-β PET scan at a participating specialty center and their care partners, from the IDEAS study. Regression models tested associations between an elevated scan result and objective and subjective economic strain outcomes. Qualitative semi-structured interviews were conducted with patients and care partners ~24-36 months post-scan occurrence.

**Results:**

Participants' mean age was 75, were majority White, non-Hispanic, highly educated, in good health, and well-resourced. Care partners were mainly spouses. Patients and care partners with elevated amyloid did not have higher economic strain at any post-disclosure time point compared to those with a negative scan. However, difficulty paying bills increased substantially for all participants over 18-24 months. Themes related to patient and care partner experiences of financial decision-making considering the scan were: 1) the need to make or update financial plans, 2) perceived care needs and financial resources for meeting care needs, and 3) involvement of family members in financial plans.

**Conclusions:**

Despite engaging in financial decision-making post-scan, participants reported experiencing economic strain, as measured by difficulty paying bills. More research is needed across the wealth distribution to develop methods for identifying and addressing economic strain experiences following a diagnosis of dementia.

**Supplementary Information:**

The online version contains supplementary material available at 10.1186/s13195-026-02010-x.

## Background

Alzheimer’s Disease or related dementia (ADRD) is associated with high costs of care from diagnosis through end of life [1, 2]. The total lifetime cost of care for a patient with ADRD in the United States is estimated at $392,874 in 2022 dollars, which is more than twice the amount incurred by individuals without ADRD [3, 4]. Despite Medicare and other sources of financial assistance, individuals with ADRD and their family members still incur high out-of-pocket expenses, due to insurance deductibles, co-payments, co-insurance, and services not covered, such as nursing home stays and other long-term care use. One study showed that the 2-year out-of-pocket medical costs more than doubled and the median wealth was reduced to less than half over the 8-year follow-up in the dementia group [5]. On average, Medicare beneficiaries with ADRD pay $10,241 out-of-pocket annually for health care and long-term care services not covered by other sources [3].

Despite the significant financial burden of ADRD, there has been minimal research to date on how a diagnosis of dementia affects current economic strain or experiences of financial planning among persons with ADRD and their care partners. An early ADRD diagnosis could reduce economic strain by enabling people with ADRD and their care partners to plan for future health and long-term care needs, including engaging family members who might provide unpaid care and communicating goals of care and preferences for long-term care. It may also prevent poor financial outcomes attributable to changes in judgment and financial decision-making capacity or financial exploitation (e.g., from scams) [6–8]. It is hoped that early engagement in financial planning or financial protections could reduce economic strain later in the disease [9]. Significant advances in using biomarkers to improve accurate and earlier diagnosis of ADRD could provide patients and care partners with information to engage in care and financial decision-making processes much earlier in the disease process. One example of a biomarker is amyloid-β positron emission tomography (PET) scans. Elevated levels of amyloid plaques are suggestive of the neuropathology associated with Alzheimer’s disease whereas non-elevated levels of amyloid may suggest the patient’s cognitive impairment is attributable to causes other than Alzheimer’s disease. Most economic evaluations estimate the costs incurred from Alzheimer’s disease, starting at the time of a clinic- or hospital-diagnosis or a cognitive score indicative of Alzheimer’s disease, until time of death [1–4, 10–12]. One recent study estimated per capita annual dementia costs of over $40,000 a year, which includes unpaid caregiving costs [10]. Few economic evaluations of dementia examine costs from the time of early diagnosis, which is a gap that we hope that this study begins to fill.

In this mixed-methods study, we aimed to: (1) explore the relationship between receipt of amyloid-β PET scan results and objective and subjective measures of economic strain from administrative and survey data, and (2) explore persons with mild cognitive impairment (MCI) or dementia and their care partners’ experiences of financial decision-making via semi-structured interviews. Combined objective and subjective economic strain data are meaningful because it is unlikely that a single definition is sufficient to capture economic strain for this population. Importantly, in addition to patients, we also obtain the perspectives of care partners in in-depth interviews, adding richness to understanding experiences of economic strain and financial decision-making beyond that of the individual with ADRD to the family system. We cautiously hypothesize that patients with elevated amyloid-β scan results and their care partners will have higher subjective economic strain post-scan and higher objective and subjective economic strain four years post-scan due to the progressive nature of ADRD and the accelerated need for more resources to manage the disease over time. Those without elevated amyloid, although also experiencing cognitive impairment, may have more uncertain progression and fewer care needs by comparison.

## Methods

### Mixed-methods study design

This is a longitudinal prospective cohort study of patient-care partner dyads enrolled in the CARE-IDEAS (Caregivers’ Reactions and Experience: Imaging Dementia Evidence for Amyloid Scanning) study. CARE-IDEAS is supplemental to the original IDEAS study conceived to examine the impact of amyloid-β PET scans on clinical outcomes in Medicare beneficiaries with MCI or dementia between 2016 and 2018. This paper used a parallel convergent mixed-methods design to explore objective and subjective experiences of economic strain among CARE IDEAS participants, where quantitative and qualitative data were collected and analyzed separately. The goal of the quantitative analysis was to enumerate potential associations between an elevated amyloid-β scan result and subsequent objective and subjective economic strain. The goal of the qualitative analysis was to explore patient and care partner perspectives and experiences of financial decision-making following the scan. The qualitative and quantitative findings were combined via narrative integration in the discussion [13].

Data sources included (1) CARE-IDEAS study variables taken during the baseline assessment (approximately 4–5 months post-PET scan results disclosure) and a follow up assessment (18–24 months post-PET scan results disclosure), (2) Medicare administrative data on Fee-for-Service and Medicare Advantage patients up to four years after the scan, and (3) qualitative interview data from patients and care partners collected between 2020 and 2021 (approximately 2–3 years post-scan).

In the following sections we first present the quantitative methods and results followed by the qualitative methods and results.

### Quantitative methods

#### Data sources and study population

The CARE-IDEAS study recruited a subsample of 2,228 IDEAS participants who had agreed to be contacted for further research opportunities, were able to provide informed consent, and were able to identify a care partner. The CARE-IDEAS study also included participation from 1,872 care partners. Participants in the IDEAS study required that a patient meet appropriate use criteria for amyloid-β PET (see more detail in Rabinovici et al., 2019) [14].

We used data from the CARE-IDEAS baseline (T1) and follow up (T2) structured telephone surveys (when available) for both the patient and the designated care partner and the 2015–2020 Medicare Master Beneficiary Summary Files (MBSF) for the patient (the beneficiary). We also used patient data on the amyloid-β PET scan results and other baseline information obtained from the parent IDEAS study. As our outcomes were derived from the Medicare administrative data and survey data, we had two cohorts: (1) the beneficiary-based cohort and (2) the survey-based cohort. Inclusion criteria for the beneficiary-based cohort required that the patient not receive the Medicare Part D low-income subsidy (LIS) or be enrolled in both Medicare and Medicaid (“dually-enrolled”) in the 12 months prior to the amyloid-β PET scan date. Inclusion criteria for the survey-based cohort required that both the patient and care partner completed the survey at T1 and responded to the perceived economic strain items at either one or both survey time points.

#### Study variables

##### Economic strain outcomes

Outcome measures were derived from Medicare administrative data and survey data as outlined above. First, we estimated via administrative data whether patients with elevated amyloid suggestive of ADRD have *higher objective economic strain*, signaled by newly enrolling in Medicaid (e.g., they became newly dually enrolled in Medicare and Medicaid) or to newly receive LIS compared to Medicare patients without an elevated scan. Medicare beneficiaries who have limited income and resources can receive a low-income subsidy for Part D (LIS). Full-benefit dual-eligible beneficiaries automatically qualify for LIS. For these two beneficiary-based economic strain outcomes, we calculated time to change in status that indicated additional economic strain as the time between the date of scan and the first day of the month of the new status, dually enrolled or receiving LIS, respectively.

Second, in terms of subjective measures, survey items ascertained whether care partners of patients with an elevated scan reported *higher perceived economic strain* compared to care partners and patients without an elevated scan. We used the three-item subscale of the Caregiver Reaction Assessment (CRA) [15] to assess subjective economic strain associated with caregiving, along with items asking both patients and care partners about perceived difficulty paying monthly bills. The three CRA subscale items asked care partners to agree or disagree, using a 5-point Likert scale (1 = disagree a lot, 5 = agree a lot), with statements asserting that 1) I have adequate financial resources for caregiving, 2) It is difficult to pay for the patient’s needs, and 3) Caring for the patient is a “financial strain”. We dichotomized these items into “not adequate” vs. “totally adequate” and “no difficulty” vs. “any difficulty”. Both patients and care partners were then asked to report the level of difficulty they experience paying their monthly bills, which we dichotomized into “no difficulty vs. “any difficulty”.

##### Exposure

The amyloid-β PET scan results were the exposure, received by all participants. Scans were interpreted by participating imaging specialists as a part of the IDEAS study using approved reading methodologies for each tracer [16–18]. Based on FDA guidelines, scans were interpreted dichotomously as “negative” (white matter retention only) or “positive” (cortical tracer retention) [14]. Elevated levels of amyloid plaques indicate neuropathology associated with Alzheimer’s disease, whereas non-elevated levels suggest that cognitive impairment is attributable to causes other than Alzheimer’s disease. Results were shared with providers who in turn informed participants and their partners. The median time between participants’ amyloid-β PET scans and the subsequent CARE-IDEAS baseline survey was 4.5 months, suggesting that most if not all participants had been informed of their scan results prior to the baseline interview.

##### Covariates

We controlled for demographic, health, and other variables assessed at T1, including sex, age, relationship of the patient to the care partner (spouse or not), education, having private LTCI, consulting a financial planner, and self-reported health status. Relevant baseline medical history variables (1 = yes, 0 = no) were pulled from clinical assessment in the IDEAS parent study data, and included atrial fibrillation, ischemic heart disease, hypertension, dyslipidemia, diabetes, active depression, history of stroke, and traumatic brain injury. These were then summed to create a count of comorbidities variable ranging from zero to eight.

#### Quantitative statistical analysis

We described the characteristics of the study cohorts using proportions for categorical variables and means with standard deviations or medians with quartiles for continuous variables grouped by amyloid-β PET scan result. We examined these characteristics separately among those without prior year LIS, without prior year dual-eligible status, and dyads with at least one timepoint of economic survey data. We presented standardized mean differences and evaluated group differences using chi-square tests for categorical variables and Kruskal-Wallis tests for continuous variables. Quantitative analyses were conducted on the Centers for Medicare and Medicaid Services Virtual Research Data Center using SAS 9.4. Missing covariate values were imputed using the mode for categorical variables, and the mean for continuous variables, with values taken from the study sample.

##### Beneficiary-based analyses

We described the rate of acquiring Medicaid (dual-enrollment) and low-income supplement (LIS) at both time points. For both the dual-eligible status and LIS economic strain outcomes, we calculated the cumulative incidence, accounting for the competing risk of death and ran separate adjusted Cox proportional hazards regression models [19] to test associations between an elevated scan result and dual-eligible status and LIS within 4 years post-scan, respectively. Due to the small number of events for both economic strain outcomes, we were only powered to include a small number of relevant covariates (age, sex, education, and race) and selected diagnoses (hypertension) in adjusted models. Patient participant censoring occurred at the earliest of the following censor points: death, end of Medicare patient data, or the end of the study period (December 31, 2020).

##### Survey-based analyses

To examine the association between patient and care partner characteristics and survey-based economic strain measures at T1 and T2, we ran separate multivariable logistic regression models with random intercepts to account for within-subject repeated measures. In separate models, we regressed CRA composite scale scores and patient and care partners’ self-reported difficulty paying monthly bills ratings at both time points (T1, T2) on scan result, time point indicators and their interactions with scan result, and adjusted for T1 covariates. Non-collinear patient covariates included medical diagnoses, age, sex, education, race, self-reported health, no LTCI, no financial planner, and comorbidity count.

The Strengthening the Reporting of Observational Studies in Epidemiology guidelines for cohort studies was used when writing this paper (See supplemental materials) [20].

### Qualitative methods

#### Recruitment

A subsample of patients and care partners who completed both survey timepoints and agreed to be contacted for future research opportunities were invited to participate in semi-structured interviews approximately 18-months after the amyloid-β scan. We oversampled patients and care partners who did not identify as non-Hispanic White to increase the diversity of perspectives. Participants were mailed an invitation to participate with a consent form before they were approached by the research team. Interviews were only conducted with participants who gave their informed consent.

#### Data collection

A total of 100 semi-structured, telephone-based interviews were completed following a topic guide. The topic guide included questions regarding participants’ experiences of getting the scan, how they felt about the result, and whether the scan result affected their future plans. The interviews were conducted by three research assistants and a research coordinator under the supervision of senior researchers. The interviewer first reviewed the consent form and then asked the participant to state the purpose of the interview, in their own words, as an additional check of capacity. Interviews lasted approximately 30 to 60 min and were recorded, with permission from the participant.

#### Qualitative analysis

The audio-recorded interview recordings were transcribed, anonymized, and imported into NVivo 12 for analysis. The qualitative data were initially organized using exploratory content analysis and double coded by five qualitative researchers (EC, EB, MSB, WZ, TW) for accuracy. All data related to patient and care partner experiences of economic strain were collated and further analyzed following Braun and Clark’s procedures for thematic analysis [21]. First, all authors reviewed the qualitative data, then the five qualitative researchers each made an initial list of codes and notes on their impressions. These codes and notes were discussed in a team meeting and used to create a consensus list of codes, which were subsequently applied to the data by two authors (EC and WZ). Examples of codes included: “Financial status at time of diagnosis”, “Aware of increasing care needs”, and “Decision to enroll in long-term care insurance”. Codes with overlapping meanings were grouped into categories. During our analysis, we noticed that participants’ expectations of the patient’s future care needs and associated financial concerns differed depending on the level of cognitive impairment and scan result. To explore this further, we used the query function in NVivo 12 to explore the data collected within the categories by the patient’s diagnosis.

## Results

### Quantitative results

#### Cohort characteristics

As shown in Table 1, the beneficiary-based cohort for the Medicaid outcome analysis (*n* = 2,076) had a mean age of 75 and was mostly White, highly educated, in good health, and well-resourced as evidenced by the high rates of having consulted a financial planner and having LTCI (cohort for LIS analysis is in Table A1). Nearly all care partners were spouses. The number of respondents in the survey-based cohort (Table 2) was slightly smaller than the beneficiary-based cohort, reflecting the inclusion criteria of the survey sample (*n* = 1,723). Characteristics were similar across the two cohorts, with the minor exception that more care partners were in fair or poor health in T1 in the surveys (12.5% versus 10%). For the survey-based cohort models, we imputed values for patient age (*N* = < 11, < 1%), patient education (*N* = 21, 1%), no financial planner (*N* = 24, 1.1%), and no long-term insurance (*N* = 123, 5.8%). As reported elsewhere [22], the mean score for the full survey-based sample on the 35-point abbreviated version of the Telephone Interview for Cognitive Status-Modified (TICS-m) was 22.0 (SD 5.7) for those with MCI which is comparable to other studies. The mean score for those with dementia was 16.6 (SD 6.0), consistent with mild dementia.

**Table 1:** Medicare-beneficiary-based cohort characteristics of patients with cognitive impairment, conditional on not having Medicaid in the year prior to survey date, by scan results at T1

Variable	Overall	Non-Elevated Scan	Elevated Scan	Std Diff	*p*-value
*N*	2,076	705	1,371		
*Patient Characteristics*					
Age, Mean (SD)	74.56 (5.55)	73.77 (5.50)	74.96 (5.54)	21.6%	< 0.001
Male	1,211 (58.3%)	419 (59.4%)	792 (57.8%)	3.4%	0.47
Care partner is spouse or significant other	1,743 (84.0%)	583 (82.7%)	1,160 (84.6%)	5.2%	0.26
Non-Hispanic White*	1,927 (92.8%)	646 (91.6%)	1,281 (93.4%)	6.9%	0.13
No college*	851 (41.0%)	296 (42.0%)	555 (40.5%)	3.1%	0.51
Has not consulted financial planner	1,203 (57.9%)	415 (58.9%)	788 (57.5%)	2.8%	0.54
Does not have long-term care insurance	1,419 (68.4%)	476 (67.5%)	943 (68.8%)	2.7%	0.56
Self-reported poor to fair general health*	350 (16.9%)	143 (20.3%)	207 (15.1%)	13.6%	0.003
*Patient Medical History*					
MCI diagnosis+ Dementia diagnosis+	1,660 (74.5%) 568 (25.5%)	692 (82.4%) 148 (17.6%)	968 (69.7%) 420 (30.3%)	0.33	< 0.001
Atrial fibrillation	188 (9.1%)	70 (9.9%)	118 (8.6%)	4.6%	0.32
Ischemic heart disease	199 (9.6%)	67 (9.5%)	132 (9.6%)	0.4%	0.93
Hypertension	1,015 (48.9%)	374 (53.0%)	641 (46.8%)	12.6%	0.007
Dyslipidemia	965 (46.5%)	339 (48.1%)	626 (45.7%)	4.9%	0.29
Diabetes	298 (14.4%)	128 (18.2%)	170 (12.4%)	16.1%	< 0.001
Active depression	379 (18.3%)	152 (21.6%)	227 (16.6%)	12.8%	0.005
History of stroke or TIA	205 (9.9%)	80 (11.3%)	125 (9.1%)	7.4%	0.11
Traumatic brain injury	112 (5.4%)	41 (5.8%)	71 (5.2%)	2.8%	0.54
Count of comorbidities, Mean (SD)	1.87 (1.52)	2.09 (1.59)	1.75 (1.47)	22.4%	< 0.001
*Care Partner*					
Self-reported poor to fair general health	214 (10.3%)	68 (9.6%)	146 (10.6%)	3.3%	0.48
General health status (self assessed), Mean (SD)	2.38 (0.89)	2.43 (0.84)	2.35 (0.91)	9.1%	0.005

This cohort was used to examine newly acquiring Medicaid (e.g., becoming a dually eligible beneficiary), conditional on not having Medicaid at the time of the T1 survey. The characteristics of the LIS sample (newly acquiring LIS), shown in Table A1, were very similar. TIA stands for transient ischemic attack

* We dichotomized education into no college (<12 years, high school graduate) vs. some college and higher (some college, Bachelor’s degree, Master’s, Professional, or Doctoral degree). Due to the highly homogenous nature of the study sample, race and ethnicity were recoded and dichotomized into non-Hispanic white vs. other (Hispanic white, non-Hispanic Black or African American, and other). Self-reported health status was dichotomized from a 5-point Likert scale into “Fair/Poor” (4=fair, 5=poor) vs. “Good+” (1=excellent, 2=very good, 3=good)

+. Dementia and MCI diagnoses were computed based on the full sample of n=2,228 as data use restrictions at the time of the revision phase prohibited us from removing individuals who were on Medicaid at baseline

**Table 2:** Survey-based economic strain cohort characteristics, perceived financial strain survey measures, by scan results at T1

Variable	Overall	Non-Elevated Scan	Elevated Scan	Std Diff	*p*-value
*N*	1,723	538	1,185		
*Patient Characteristics*					
Age, Mean (SD)	74.47 (5.49)	73.65 (5.47)	74.85 (5.46)	21.9%	< 0.001
Male	1,050 (60.9%)	346 (64.3%)	704 (59.4%)	10.1%	0.05
Care partner is spouse or significant other	1,524 (88.5%)	474 (88.1%)	1,050 (88.6%)	1.6%	0.76
Non-Hispanic White*	1,603 (93.0%)	496 (92.2%)	1,107 (93.4%)	4.7%	0.35
No college*	699 (40.6%)	225 (41.8%)	474 (40.0%)	3.7%	0.48
Has not consulted financial planner	991 (57.5%)	312 (58.0%)	679 (57.3%)	1.4%	0.79
Does not have long-term care insurance	1,168 (67.8%)	368 (68.4%)	800 (67.5%)	1.9%	0.71
Self-reported poor to fair general health*	285 (16.5%)	108 (20.1%)	177 (14.9%)	13.6%	0.008
*Patient Medical History*					
Atrial fibrillation	155 (9.0%)	56 (10.4%)	99 (8.4%)	7.1%	0.17
Ischemic heart disease	168 (9.8%)	51 (9.5%)	117 (9.9%)	1.3%	0.80
Hypertension	860 (49.9%)	299 (55.6%)	561 (47.3%)	16.5%	0.002
Dyslipidemia	803 (46.6%)	264 (49.1%)	539 (45.5%)	7.2%	0.17
Diabetes	246 (14.3%)	94 (17.5%)	152 (12.8%)	13.0%	0.01
Active depression	308 (17.9%)	114 (21.2%)	194 (16.4%)	12.4%	0.02
History of stroke or TIA	175 (10.2%)	63 (11.7%)	112 (9.5%)	7.3%	0.15
Traumatic brain injury	94 (5.5%)	30 (5.6%)	64 (5.4%)	0.8%	0.88
Count of comorbidities, Mean (SD)	1.88 (1.53)	2.14 (1.61)	1.76 (1.48)	24.2%	< 0.001
*Care Partner*					
Self-reported poor to fair general health	212 (12.3%)	68 (12.6%)	144 (12.2%)	1.5%	0.78
General health status (self assessed), Mean (SD)	2.37 (0.96)	2.44 (0.96)	2.34 (0.97)	9.6%	0.05

*TIA* stands for transient ischemic attack

*All covariates were taken from T1 survey data. We dichotomized education into no college (<12 years, high school graduate) vs. some college and higher (some college, Bachelor’s degree, Master’s, Professional, or Doctoral degree). Due to the highly homogenous nature of the study sample, race and ethnicity were recoded and dichotomized into non-Hispanic white vs. other (Hispanic white, non-Hispanic Black or African American, and other). Self-reported health status was dichotomized from a 5-point Likert scale into “Fair/Poor” (4=fair, 5=poor) vs. “Good+” (1=excellent, 2=very good, 3=good). For the survey-based cohort models, we imputed values for patient age (*N*=<11, <1%), patient education (*N*=21, 1%), no financial planner (*N*=24, 1.1%), and no long-term insurance (*N*=123, 5.8%)

There were some key differences in patients in the beneficiary-based cohort who received an elevated (1,371) versus non-elevated scan result (*n* = 705). Individuals with an elevated scan were older (by 1 year on average) and healthier. Rates of some diagnosed conditions were lower in the elevated scan patient group, including hypertension, diabetes, active depression, and a lower mean count of comorbidities. Care partners characteristics did not meaningfully differ by patient scan results (Table 1).

#### Beneficiary-based economic strain analyses outcomes

The cumulative incidence rates show that the number of patients who became newly eligible for Medicaid was extremely low 4 years after the baseline survey date (1.3%) and was similar across scan results (elevated: 1.1%, non-elevated: 1.8%) (Figure A1). Similarly, newly receiving LIS was slightly more common than newly receiving Medicaid (e.g., dually covered), reflecting looser eligibility requirements for LIS, but incidence was still extremely low. Just 44/2,076 patients at 4 years were newly eligible for LIS, representing 2.4% of the patient sample. Again, incidence rates were similar between scan groups (elevated: 2.8%, non-elevated: 1.8%).

The adjusted hazard ratios from the Cox proportional models also show no significant association between scan result (elevated vs. not elevated) and time to eligibility for Medicaid status or LIS (Table 3).

**Table 3:** Cox model results for incident Medicaid and Low-Income Subsidy status up to four years after the initial scan date

	Unadjusted		Adjusted	
	HR (95% CI)	*p*	HR (95% CI)	*p*
Outcome = dual-eligible status				
Elevated scan v. not elevated	1.39 (0.71, 2.70)	0.33	1.20 (0.60, 2.43)	0.60
Outcome = LIS				
Elevated scan v. not elevated	0.56 (0.26, 1.24)	0.15	0.49 (0.21, 1.11)	0.09

#### Survey-based economic strain analyses outcomes

**Fig. 1:**
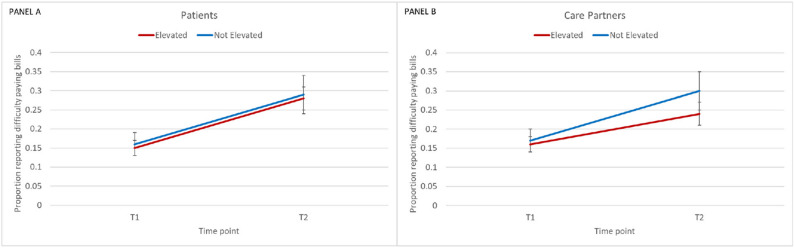
Model-estimated proportions by scan and time point on difficulty paying monthly bills for patients (Panel **A** on LHS) and for care partners (Panel **B** on RHS). Note: The increase over time was significant for each group (*p*<0.05)

None of the subjective economic strain outcomes differed by scan result. However, we observed that, on average, the full sample of patients and care partners expressed greater difficulty paying bills at T2 than T1 (Fig. 1, Panels A and B). The increase in having at least some difficulty paying bills was relatively large in magnitude, going from 17 to 19% of patients who expressed difficulty paying bills at T1 to 28–34% at T2 (Fig. 1, Panel A and Table A2). The difficulty of paying bills was also significantly higher for care partners at T2 (from 18 to 20% to 24–33% in T2) (Fig. 1, Panel B and Table A2).

Covariates included were diagnosis (MCI or dementia), patient age, patient sex and patient education (no college, vs. college or more), patient race, patient self-reported health (binary variable), care partner work status, no long-term insurance, no financial planner, and patient comorbidity count.

Sample is from the administrative data cohort, limited to those who did not have Medicaid or LIS, respectively, in 12 months prior to scan date, Covariates included were diagnosis (mild cognitive impairment or dementia), patient age, sex, education (no college vs. college or more), and race.

### Qualitative results

We interviewed 38 patients and 62 care partners (30% of patients and care partners were complete dyads). Table A4 provides an overview of the characteristics of the qualitative sample of participants. We identified three themes relating to patient and care partner experiences of financial decision-making following an amyloid-β scan in light of the scan results: (1) the need to make or update financial plans, (2) perceived care needs and financial resources for meeting care needs, and (3) involvement of family members in financial decision-making. See Table A5 for illustrative quotes within these identified themes.

#### Making or updating financial plans

Many participants reported making plans for their care in older age (e.g., drafting wills, enrolling in LTCI, and consulting financial planners) before receiving the amyloid-β scan. Participants with MCI without elevated amyloid acknowledged the existence of such plans, should they be needed in the future, but did not report updating them or making new ones. Participants with MCI and elevated amyloid reported the scan result gave them a better understanding of what to expect for their future care needs. They described updating their plans or making new ones in light of the scan result:

*“We made sure we already had wills and things*,* but we made sure to update them. We got our affairs in order. We went through everything together*,* all of our finances*,* everything together. I remember shortly after the diagnosis*,* we spent a couple of weeks together in the basement going through everything so that [we’d] both be aware of where everything was.” (Care partner*,* MCI with elevated amyloid)*.

And:

*“[The scan] helped me strategize and make plans*,* and get all the financial and legal … new wills and all of those things organized. So yeah*,* it’s been a very*,* very useful thing.” (Patient*,* MCI with elevated amyloid)*.

On the other hand, one participant described how the results from the amyloid-β scan prompted them to engage in financial decision-making, specifically related to estate planning, but was prohibited by the cost of a lawyer:

*“Well*,* the process is*,* we had it done*,* and now I’m waiting to get some money from my 401k so I can pay for the attorneys to [write up a will]. It’s so expensive. That’s where I’m at right now.” (Patient*,* MCI with elevated amyloid)*.

#### Perceived care needs and financial resources for meeting care needs

Participants described their anticipated care needs after receiving the scan results and how they would meet these expected costs. For example, participants with dementia mainly described needing nursing home care in the future and discussed how they would pay for this. Some expressed concerns that they would live in a nursing home longer than their economic resources would allow for: “I don’t need much. We’re fairly comfortable, so I don’t have any financial fears. My only fear is living too long and spending all that money in a nursing home.” (Patient, Dementia without elevated amyloid).

Participants discussed how they would leverage their economic resources (including Medicare, LTCI, retirement savings, support from children, and their personal wealth) to meet their future care needs. Participants who described having the resources to meet their anticipated care needs reported feeling they had control over their future care. They described having flexibility, contingencies, and the ability to take things as they come: “But, we do have a financial advisor who has worked with us, and we’ve got several financial buckets. And we’ve got care. And if it comes down to it, we’ll sell our house.” (Care partner, MCI with elevated amyloid). Participants who reported not having enough resources to meet their future care needs were worried about how they would pay for future care:

*“I know*,* if it gets bad enough*,* I’ll end up in a nursing home. That’s one area that I don’t have financial stability on. I do not have one of those home care policies or anything or not home care but nursing home policy… I’ll probably have to go on [Medicaid] and whatever facility they put me in.” (Patient*,* MCI with elevated amyloid)*.

Whether the person with cognitive impairment had LTCI was an important factor in whether participants reported having the resources for meeting their future care needs. Often, this decision was made long before the onset of symptoms:

*“The problem is long ago when we purchased her long-term care insurance … This is a real serious thing for us … We had the chance to get a kind of a 10% family discount if we both got the insurance. And I foolishly said*,* ‘Well*,* I don’t need to yet.’ This is before the mild cognitive impairment was clear. So as soon as I took my first pill of Aricept*,* I became poison to the insurance companies*,* and I am uninsurable.” (Patient*,* MCI without elevated amyloid)*.

#### Involvement of family members in financial decision-making

Participants talked about how financial decisions regarding future care could affect their families. Some participants decided to keep discussions about their care needs within the caregiving dyad, whereas some sought to involve others. If participants involved others in financial decisions, they sought to involve people who they perceived as trustworthy and knowledgeable. Participants sometimes sought relatives or close friends to act as trustees or executors to the participants’ finances: “Well, I don’t really discuss the planning with my children. I keep them up to date. They’re adults, but occasionally I talk with my wife about it and it sort of is a factor in our financial planning.” (Patient, MCI without elevated amyloid).

While some participants noted the importance of involved, trusted individuals (e.g., children) in financial plans, they were also concerned their future care needs and associated costs could negatively impact their family. These concerns included becoming a financial “burden” in the later stages of dementia and diminishing their family’s shared wealth. Participants specifically cited concerns about accumulating debt or diminishing the inheritance intended for their children or spouse:

*“Well*,* like most people*,* I just don’t want to be a burden on my family*,* especially with kids. We’re working very hard to make sure that we’re debt-free and we actually have been giving the kids things*,* so that when the time comes*,* they already have things we want them to have and they don’t have to go through a lot of probate and all that sort of stuff.” (Patient*,* MCI with elevated amyloid)*.

### Integrated quantitative and qualitative findings

In this mixed-methods study, we explored patient and care partner experiences of economic strain and financial decision-making following an amyloid-β PET scan. In our analysis of quantitative data, we found low levels of objective economic strain in this cohort, as indicated by very low rates of Medicaid status (1–2%) and LIS (2–3%) in both post-scan time periods. We also found no association between scan result and subsequent subjective economic strain. However, subjective outcomes from survey data indicated that substantially more participants reported at least some difficulty with paying bills around 18 months after the scan compared to around 4 months after the scan, irrespective of the scan result. Our qualitative analysis suggests that experiences of economic strain may be nuanced. For example, receiving an elevated amyloid-β scan result influenced participants’ expectations for their future care needs and prompted them to engage in financial decision-making. These participants expected to need potentially costly nursing home care. Those who did not report having the resources to pay for their perceived future care described being worried about their financial stability in the future. They also described concerns that paying for their future care would have a negative economic impact on their family. Taken together, these findings indicate that amyloid-β scans can shape patient and care partner expectations of their future care needs and costs, which can inform financial decision-making, but may not translate into changes in objective or subjective measures of economic strain.

## Discussion

As noted, we did not find any differences in quantitative measures of objective and subjective economic strain by scan result. There are several possible explanations for this finding. First, patients and care partners in this study were highly educated and over half had consulted a financial planner and/or purchased LTCI, and therefore were unlikely to experience incident economic strain following the scan result. Additionally, our administrative-based measures in particular (newly qualifying for Medicaid or Medicare Part D LIS), represented a catastrophic change in financial security, and therefore may be a rare outcome. Subjective strain, by contrast, increased over time regardless of scan result, with much higher rates of individuals reporting difficulty paying bills. Second, because all participants had MCI or dementia, it is unclear what the added benefit of knowing the cause of their memory problems from the amyloid-β PET scan when considering financial planning, although understanding etiology may be important for future planning. Third, participants with non-elevated levels of amyloid were in poorer health than participants with elevated amyloid both in terms of self-reported general health and the number of comorbid conditions, including depression [23]. Therefore, other factors may explain the lack of relationship between the scan results and economic strain. Specifically, it is possible that increases in objective and subjective economic burden among dyads with elevated amyloid are driven by increasing care needs and costs associated with progressive ADRD, whereas both objective and subjective burden among dyads with non-elevated amyloid are driven by poor general health and multimorbidity. Participants did discuss some current costs. Overall, however, the qualitative results revealed anxiety about future costs related to nursing home care and other unknown costs, even for people with economic resources.

Findings from our qualitative analysis highlight that the scan result prompted many patients with elevated amyloid and their care partners to make or update financial or future care plans. This is in contrast to previous qualitative research from the United Kingdom (UK), which found that people with dementia and their care partners were reluctant to make financial plans due to feelings of uncertainty regarding the future and put off engaging in financial decision-making [24]. It is possible that a diagnosis in light of an amyloid-β scan result gave patient and care partners greater certainty about future care needs than a diagnosis without a scan, even at the MCI stage of disease, which our study included but the UK study did not, or that our study reflected worries for the future rather than actual financial strain given we had people with MCI included. Participants in this study described how they would leverage existing financial resources to pay future long-term care costs. However, participants who reported having inadequate resources expressed concerns about how they would meet the costs of their future care. Participants discussed how their future care needs and associated costs may affect their wider family unit. Patients specifically cited concerns that they might financially “burden” their families and that paying for their care would deplete their savings and family wealth. Similar concerns have been reported by people with dementia and their caregivers in the UK [25]. Giebel et al. also noted that both participants with dementia and their care partners who had the resources to make advanced financial plans for their care were satisfied with arrangements for their future care [25]. Similarly, participants in this study who reported having the financial resources necessary to meet the patient’s care needs described having the flexibility to “take things as they come.” Despite many participants reporting engaging in financial decision-making or describing having the resources to meet the patient’s anticipated care needs, participants still experienced increased difficulties with paying their bills. It is possible that engaging in financial decision-making or feeling able to meet future care needs is not sufficient for preventing economic strain.

As the prevalence of ADRD continues to grow rapidly, it is imperative to understand and minimize the economic impacts on those living with the disease and their care partners. To date, there has been limited research on both objective and subjective economic strain experienced by patients and their care partners, particularly at the time surrounding early diagnosis. While our study strived to fill that gap by identifying and using multiple measures of objective and subjective economic strain, more research is needed to elucidate how best to define and measure the economic impacts of ADRD on patients and care partners. For example, our study showed that “difficulty paying bills” resonated with patient and care partner perceptions of economic strain, potentially because bills are a recurring cost to them, and it appeared more sensitive than other measures. Although promising, a wider range of measures are needed to reflect the multifaceted and evolving nature of economic strain highlighted in our study and to capture the heterogeneous disease journey experienced by patients with ADRD and their care partners. These efforts are particularly important given the evidence that deterioration in financial capacity is an early indicator for ADRD [26]. Measures of both objective and subjective strain could be integrated into the intake or diagnostic work-up questionnaires used by primary and geriatric care providers or within the electronic health record (EHR). Documenting both health and financial concerns at the time of diagnosis of MCI or dementia and throughout the ADRD continuum could help connect patients and care partners with available resources. Best practices could be gleaned from work integrating screening for the social determinants of health and financial toxicity associated with cancer treatment into routine care [27, 28].

Relatedly, it has been proposed that early diagnosis of ADRD informed by an amyloid-β PET scan can motivate advanced care and financial decision-making, which can reduce economic burden for patients and care partners. Many of our interviewees noted that an elevated scan spurred consideration of and action on their financial matters, or they derived comfort that those matters had been set in motion or established earlier. Patients and care partners with limited financial means or non-financial support to access these resources before or following diagnosis may be placed at even greater risk for economic strain. Therefore, more research and practice, and policy innovation are needed to identify, test, and implement advanced care and financial decision-making strategies to address the diverse economic support needs of patients and care partners and ensure equitable access to these critical resources. Potential actions to be explored could include leveraging health systems as a point of contact for these patients, including the use of financial navigators; coverage of advanced care and financial planning in Medicare and Medicaid; provider and other health professional education on available resources; and, leveraging community-based resources, such as the Area Agencies on Aging or other state-funded, dementia support for patients and care partners.

### Limitations

Our study has several limitations. First, this sample is affluent and participants began the study with extremely low objective economic strain, which means they are not representative of the general Medicare population. Consequently, we have a sample that is expected to be among the most resilient to economic strain following an amyloid-β PET scan and therefore less likely at risk for the objective measures of economic strain, such as LIS or Medicaid eligibility. Second, our quantitative data almost entirely comprised persons who identify as White, non-Hispanic, which is a major limitation given racial inequality in economic strain in the United States. The subset of participants who completed the qualitative interviews was more racially diverse, but this does not overcome the problem. Third, to join the study, individuals had to find their way to a specialized center with scan technology, have an atypical case (e.g., progressive, unexplained MCI or dementia of uncertain cause), and provide informed consent for research (that is, these participants were not receiving routine medical care). Fourth, for the subjective strain survey data measures, we do not have reports of strain prior to receiving the scan result, as the first survey was delivered after the scan results were likely communicated to patients and care partners. As such, from the survey we have short-term economic strain (4–6 months) following scan result, and longer-term economic strain (18–24 months). We have even longer-term economic strain in the administrative data (e.g., Medicaid status at 4 years), however, economic strain may have arisen later in the disease trajectory and beyond our study time frame, or included in the time frame, for example, if individuals engaged in any (unobserved) strategic spend down to qualify for Medicaid or had more rapid cognitive decline. Fifth, we are unable to distinguish type of dementia (e.g., vascular, Lewy body, frontotemporal) and it may be that the outcome of the amyloid PET scan may not indicate differential symptom trajectories for individuals with progressive dementing conditions, or even that associated emerging comorbidities in the non-elevated group could contribute to economic costs in ways that masked any differences directly related to scan status. Sixth, we found elsewhere that patients and care partners are relatively concordant in understanding the results and their implications [22], and qualitative data also showed, with some exceptions, a relatively good understanding of what the PET scan meant for the future. And yet, we do not know how information was communicated to participants. It is possible that they were not told that the course of their decline may not change much based on their PET scan results. Finally, unlike the survey, in-depth interviews occurred during the pandemic, which may have shaped perceptions of financial strain. Also, given qualitative data collection occurred last, more individuals likely progressed from MCI to dementia than in the survey data; these individuals may have been unable to participate in the qualitative interviews or were represented through their care partner’s perspectives.

Keeping the selectivity of the study participants in mind, the uniqueness of the economic strain data obtained following a test for early diagnosis (amyloid-β PET scan) holds value for informing future examinations of economic strain. Prior to this study, we knew very little about how diagnosis following an amyloid-β PET scan (or early diagnosis from biomarkers in general) related to economic strain of persons with memory problems, including how economic strain changed over time. This study provides critical preliminary evidence to fill this gap by employing a variety of quantitative and qualitative economic strain measures and collecting multiple perspectives from both patients and care partners. Given that early diagnosis efforts will continue to quickly improve and be scaled out to large populations (e.g., Amyvid scan) [29], collecting economic strain data will remain an important strategy to help meet the needs of patients and families from day of diagnosis onward.

## Conclusions

In a sample of Medicare beneficiaries with MCI or dementia, we did not find support for our hypothesis that patients and care partners with elevated amyloid based on scan results would have higher subjective economic strain immediately post-scan nor higher subjective and objective economic strain 18–24 months and 4 years post-scan, respectively, compared to those with a negative scan. However, difficulty paying bills increased substantially for all participants on average over 18–24 months and the interviews indicated the scan prompted participants to make financial plans for future care. Furthermore, the qualitative data revealed that economic strain was very nuanced for this study sample, including revealing worries about today and tomorrow. Participants who did not report having the financial resources to meet their anticipated care needs were worried about the future. An early diagnosis for cognitive impairment could provide an opportunity to engage in financial decision-making and address existing and future financial burdens for patients and care partners. More efforts are needed to ascertain how economic strain is experienced by diverse patients and care partners and how best to measure and address it across the ADRD continuum.

## Supplementary Information

Supplementary Material 1.

Supplementary Material 2.

## Data Availability

The data used in this study from the Centers for Medicare and Medicaid Services is not publicly available.  They were accessed under the strict terms of a CMS data use agreement (DUA) that prohibits the release of data at the person level no matter the level of identifiability and limits the amount of aggregated data that can be released. Researchers interested in replicating the results of these analyses may enter into their own DUA with CMS.  See the Research Data Assistance Center (ResDAC) at [www.resdac.org] for assistance. Non-CMS data used in this study may be available from the authors upon reasonable request. Codebooks and syntax are available in the Brown Digital Repository at[https://doi.org/10.26300/ffba-3 × 69].

## Abbreviations

AD: Alzheimer’s disease
ADRD: Alzheimer’s disease and related dementias
CARE-IDEAS: Caregivers’ reactions and experience: imaging dementia–evidence for amyloid scanning
CRA: Caregiver reaction assessment
DUAL: Dual eligible for medicaid and medicare
IDEAS: Imaging dementia–evidence for amyloid scanning
LIS: Low income subsidy for part D medicare
LTCI: Long-term care insurance
MCI: Mild cognitive impairment
PET: Positron emission tomography
EHR: Electronic health record
MBSF: Master Beneficiary Summary File
TCIS-m: Telephone Interview for Cognitive Status – modified
