# Supplementary material for: Amyloid-β PET scans, economic strain and financial decision-making among persons with cognitive impairment and care partners: a mixed-methods analysis of the CARE-IDEAS study
Source: Alzheimers Res Ther. 2026 Apr 14;18:125. doi: 10.1186/s13195-026-02010-x (PMC13195842; doi:10.1186/s13195-026-02010-x)
Supplement: Supplementary file 2 — Supplementary Material 2. [file 13195_2026_2010_MOESM2_ESM.docx]

**Appendix.**

**APPENDIX TABLE A1**

**Table A1. Medicare-beneficiary-based cohort characteristics of patients with cognitive impairment, among those with no Part D Low Income Subsidy (LIS) in the prior year of the survey date, by scan result**

| **Variable** | **Overall** | **Non-elevated Scan** | **Elevated Scan** | **Std Diff** | **p-value** |
| --- | --- | --- | --- | --- | --- |
| N | 2,059 | 696 | 1,363 |  |  |
|  |  |  |  |  |  |
| Patient Characteristics |  |  |  |  |  |
| Age, Mean (SD) | 74.57 (5.55) | 73.79 (5.51) | 74.97 (5.54) | 21.4% | < .001 |
| Male | 1,205 (58.5%) | 415 (59.6%) | 790 (58.0%) | 3.4% | .47 |
| Care partner is spouse or significant other | 1,735 (84.3%) | 580 (83.3%) | 1,155 (84.7%) | 3.8% | .41 |
| Non Hispanic white | 1,911 (92.8%) | 638 (91.7%) | 1,273 (93.4%) | 6.6% | .15 |
| No college | 840 (40.8%) | 291 (41.8%) | 549 (40.3%) | 3.1% | .50 |
| Has not consulted financial planner | 1,187 (57.6%) | 407 (58.5%) | 780 (57.2%) | 2.5% | .59 |
| Does not have long-term care insurance | 1,408 (68.4%) | 469 (67.4%) | 939 (68.9%) | 3.2% | .49 |
| Self-reported poor to fair general health | 341 (16.6%) | 138 (19.8%) | 203 (14.9%) | 13.1% | .004 |
|  |  |  |  |  |  |
| Patient Medical History |  |  |  |  |  |
| MCI diagnosis +  Dementia diagnosis + | 1,660 (74.5%)  568 (25.5%) | 692 (82.4%)  148 (17.6%) | 968 (69.7%)  420 (30.3%) | 0.33 | < .001 |
| Atrial fibrillation | 187 (9.1%) | 69 (9.9%) | 118 (8.7%) | 4.3% | .35 |
| Ischemic heart disease | 193 (9.4%) | 63 (9.1%) | 130 (9.5%) | 1.7% | .72 |
| Hypertension | 1,002 (48.7%) | 368 (52.9%) | 634 (46.5%) | 12.7% | .006 |
| Dyslipidemia | 957 (46.5%) | 335 (48.1%) | 622 (45.6%) | 5.0% | .28 |
| Diabetes | 295 (14.3%) | 126 (18.1%) | 169 (12.4%) | 15.9% | < .001 |
| Active Depression | 375 (18.2%) | 150 (21.6%) | 225 (16.5%) | 12.9% | .005 |
| History of stroke or TIA | 202 (9.8%) | 79 (11.4%) | 123 (9.0%) | 7.7% | .09 |
| Traumatic brain injury | 112 (5.4%) | 41 (5.9%) | 71 (5.2%) | 3.0% | .52 |
| Count of comorbidities, Mean (SD) | 1.86 (1.51) | 2.09 (1.59) | 1.74 (1.46) | 22.7% | < .001 |
|  |  |  |  |  |  |
| Care Partner |  |  |  |  |  |
| Self-reported poor to fair general health | 213 (10.3%) | 68 (9.8%) | 145 (10.6%) | 2.9% | .54 |
| General health status (self assessed), Mean (SD) | 2.38 (0.89) | 2.43 (0.85) | 2.35 (0.91) | 9.7% | .004 |

Note: This cohort was used to examine the association of scan results with LIS, among those patient participants who did not have LIS at T1

+. Dementia and MCI diagnoses were computed based on the full sample of n=2,228 as data use restrictions at the time of the revision phase prohibited us from removing individuals who were on Medicaid at baseline.

**Figure A1.** **Cumulative incidence curves, with rates accounting for the competing risk of death for (A) Part D low-income subsidy (LIS), and (B) Dual Medicare/Medicaid status, for up to 4 years after the date of the Amyloid- β PET Scan.**


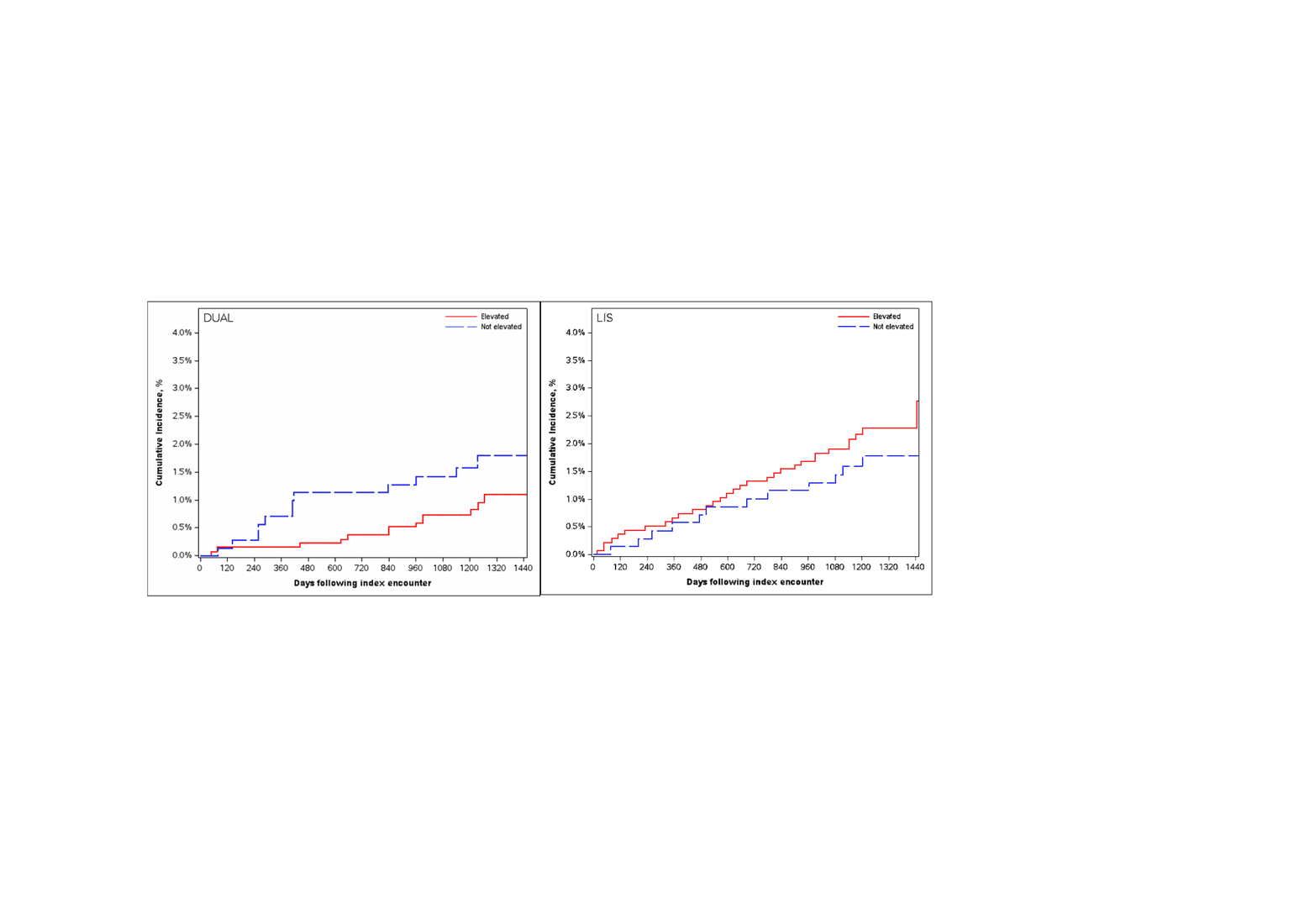


**Figure A2. Model-estimated proportion of Care Partners reporting a Caregiver Reaction Assessment Financial Strain sub-score greater than 1 from the survey-data, by scan and time point.**


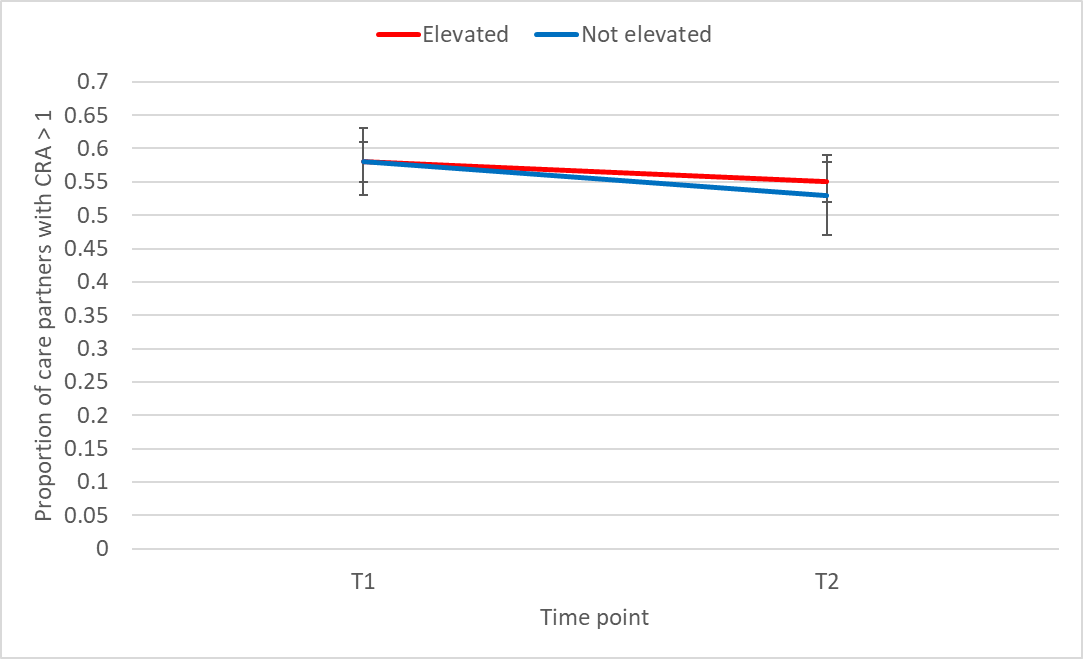


Covariates included were diagnosis (MCI or dementia), patient age, patient sex and patient education (no college, vs. college or more), patient race, patient self-reported health (binary variable), care partner work status, no long-term insurance, no financial planner, and patient comorbidity count.

Note: A CRA score greater than 1 indicates endorsement of some level of financial strain on at least one component question.

**Table A2. Survey-based financial strain outcomes, by scan and time.**

|  | **TIME 1** | | | |  | **TIME 2** | | | |
| --- | --- | --- | --- | --- | --- | --- | --- | --- | --- |
| **Patient Variables** | **Overall** | **Non-Elevated** | **Elevated** | **Std Diff** | **Overall** | **Non-Elevated** | **Elevated** | | **Std Diff** |
| N | 1,684 | 528 | 1,156 |  | 935 | 316 | 619 |  | |
| Difficulty meeting monthly bill payments | 294 (17.5%) | 102 (19.3%) | 192 (16.6%) | 7.1% | 278 (29.7%) | 106 (33.5%) | 172 (27.8%) | 12.5% | |
|  |  |  |  |  |  |  |  |  | |
| **Care Partner Variables** |  |  |  |  |  |  |  |  | |
| N | 1,718 | 537 | 1,181 |  | 1,246 | 382 | 864 |  | |
| Difficulty meeting monthly bill payments | 315 (18.3%) | 106 (19.7%) | 209 (17.7%) | 5.2% | 330 (26.5%) | 124 (32.5%) | 206 (23.8%) | 19.3% | |
| N | 1,699 | 534 | 1,165 |  | 1,200 | 534 | 1,165 |  | |
| Financial resources not adequate* | 795 (46.8%) | 253 (47.4%) | 542 (46.5%) | 1.7% | 499 (41.6%) | 161 (44.8%) | 324 (39.7%) | 10.5% | |
| N | 1,709 | 535 | 1,174 |  | 1,325 | 535 | 1,174 |  | |
| Difficult to pay for patient needs* | 654 (38.3%) | 205 (38.3%) | 449 (38.2%) | 0.1% | 427 (32.2%) | 140 (33.4%) | 280 (31.1%) | 4.9% | |
| N | 1,708 | 533 | 1,175 |  | 1,211 | 533 | 1,175 |  | |
| Caring for the patient is financial strain* | 468 (27.4%) | 138 (25.9%) | 330 (28.1%) | 4.9% | 326 (26.9%) | 98 (27.1%) | 224 (26.9%) | 0.5% | |
| N | 1,685 | 530 | 1,155 |  | 1,174 | 356 | 818 |  | |
| Financial Strain score > 1 | 966 (57.3%) | 312 (58.9%) | 654 (56.6%) | 4.5% | 627 (53.4%) | 190 (53.4%) | 437 (53.4%) | 0.1% | |

* Financial strain score is constructed from sum of these three item

Table A3. Associations of financial strain, scan, and time from adjusted models.

| **Label** | **CRA score > 1** | **Difficulty paying monthly Bills  Patient** | **Difficulty paying monthly Bills  Care Giver** |
| --- | --- | --- | --- |
| Odds Ratios : |  |  |  |
| T1 v T2, elevated | 0.83 (0.49, 1.16) | 2.08 (1.10, 3.07) | 1.97 (1.11, 2.83) |
| T1 v T2, non-elevated | 0.84 (0.63, 1.05) | 2.18 (1.56, 2.81) | 2.12 (1.60, 2.64) |
| elevated v non-elevated at T1 | 0.98 (0.75, 1.21) | 0.95 (0.69, 1.21) | 0.93 (0.68, 1.18) |
| elevated v non-elevated at T2 | 1.06 (0.77, 1.35) | 0.94 (0.65, 1.24) | 0.72 (0.52, 0.92) |
| Marginal Effects: |  |  |  |
| T1 v T2, elevated | -0.02 (-0.06, 0.02) | 0.13 (0.09, 0.21) | 0.08 (0.05, 0.11) |
| T1 v T2, non-elevated | -0.04 (-0.10, 0.02) | 0.15 (0.09, 0.21) | 0.14 (0.09, 0.19) |
| elevated v non-elevated at T1 | 0.00 (-0.06, 0.05) | -0.00 (-0.05, 0.03) | -0.01 (-0.05, 0.03) |
| elevated v non-elevated at T2 | 0.01 (-0.05, 0.08) | -0.00 (-0.08, 0.06) | -0.07 (-0.13, -0.01) |

Covariates included were diagnosis (MCI or dementia), patient age, patient sex and patient education (no college, vs. college or more), patient race, patient self-reported health (fair/poor vs. good or better), care partner work status, no long-term insurance, no financial planner, and patient comorbidity count.

Note: Marginal effects represent differences in proportions.

Table A4. Characteristics of qualitative sample for financial strain analysis

|  | Patients | Care partners |
| --- | --- | --- |
| Characteristic | N = 38 | N = 62 |
| Age, Mean (SD) | 73.5 (5.1) | 69.5 (9.1) |
| Male (%) | 25 (65.8) | 15 (24.2) |
| Caring for/by a spouse (%) | 33 (86.8) | 54 (87.1) |
| Non-Hispanic, White (%) | 25 (67.6) | 34 (54.8) |
| No college (%) | 10 (27.0) | 17 (28.8) |
| Has or caring for patient with MCI (%) | 35 (92.1) | 49 (79.0) |
| Has or caring for patient with elevated amyloid (%) | 23 (60.5) | 36 (58.1) |
| Has not consulted financial planner (%) | 19 (54.3) | - |
| Does not have long-term care insurance (%) | 24 (63.2) | - |

**Table A5. Themes relating to patient and care partner experiences of economic strain with supporting quotes**

| **Theme** | **Supporting quotes** |
| --- | --- |
| The need to make or update financial plans in light of the scan result | “We made sure we already had wills and things, but we made sure to update them. We got our affairs in order. We went through everything together, all of our finances, everything together. I remember shortly after the diagnosis, we spent a couple of weeks together in the basement going through everything so that we both be aware of where everything was. We just got everything in order.” (Care partner, MCI with elevated amyloid)  “We got an elder lawyer and we did our living wills, our medical power of attorney. We did our regular will with everything, so that everything is above board and solved” (Care partner, MCI without elevated amyloid)  “At the onset, when we got the results, we did go up to a central point where everybody met and we gave everybody the news and we went to his lawyer and revised his will and power of attorney and surrogate, everything.” (Care partner, MCI with elevated amyloid)  “[The scan] helped me strategize and make plans, and get all the financial and legal ... new wills and all of those things organized. So yeah, it's been a very, very useful thing.” (Patient, MCI with elevated amyloid)  “Well, the process is, we had it done, and now I'm waiting to get some money from my 401k so I can pay for the attorneys to [write up a will]. It's so expensive. That's where I'm at right Now” (Patient, MCI with elevated amyloid) |
| Perceived care needs and financial resources for meeting care needs | “[The scan results] just mean I know what to look forward to. I know what's going to happen. I'm 77 years old, so I know that some plans need to be made for me about what my care will be. My husband who's my caregiver right now for the things that I need, and it depends on how long he lives. I would hate to be on a Medicare nursing home.” (Patient, MCI with elevated amyloid)  “I don't need much. We're fairly comfortable, so I don't have any financial fears. My only fear is living too long and spending all that money in a nursing home, when I'd just as well be in the grave.” (Patient, dementia without elevated amyloid)  “We are fortunate in that have a very good health insurance in addition to Medicare, and we're able to choose our own doctors. And we have a team. Each of us have a team of people who are at the top of the field and in whom I have the greatest confidence and were I to develop you name it, I would either get a recommendation from an appropriate member of our medical team.” (Patient, MCI without elevated amyloid)  “Well, we have our plan for our care needs pretty much set up, in the sense that my husband was not able to get long-term care insurance, although I do have it. And so we have saved money to pay for that. Our savings are sitting there, and we are waiting to use them if we need to.” (Care partner, MCI without elevated amyloid”  “I like that he doesn't need much care yet, his pain has increasing, but he's not on any narcotics yet. So I like that he's comfortable in his own home. And I have a friend who has experienced taking care of people in end of life situations, and I've lined her up to help as needed. So I have the resources I need and I have the funds to pay for it. So, that's what I like a lot of people don't.” (Care partner, MCI with elevated amyloid)  “I know, if it gets bad enough, I'll end up in a nursing home. That's one area that I don't have financial stability on. I do not have one of those home care policies or anything or not home care but nursing home policy.” (Patient, MCI with elevated amyloid)  “Well, obviously it would depend on what that is and what the needs are. We'll do the right thing. We have proper insurance. We have the ability to be flexible.” (Patient, MCI with elevated amyloid)  “While my husband was still alive and I had an opportunity to do so, I took out long-term care insurance several years before all of this blew up. But at the time I thought, ‘You know, I think I will go with five years instead of three, even though it's more expensive.’ Because if I would get into a situation where it was mental more than physical... Most people that go in the nursing home only are there under two years, but not if it's this type of an issue. So I got the five years, so it would hopefully take the brunt of it. I'm glad I made that decision. I hope I never have to use it. I'd love to have the insurance company make money on me” (Patient, MCI, without elevated amyloid)  “I do not have one of those home care policies or anything or not home care but nursing home policy. By the time it came out I was too old to get it at a decent price, and so I never got it. I'm not financially wealthy enough to just pay for it for years.” (Patient, MCI with elevated amyloid)  “The problem is long ago when we purchased her long-term care insurance ... This is a real serious thing for us ... We had the chance to get a kind of a 10% family discount if we both got the insurance. And I foolishly said, "Well, I don't need to yet." This is before the mild cognitive impairment was clear. So as soon as I took my first pill of Aricept, I became poison to the insurance companies, and I am uninsurable.” (Patient, MCI without elevated amyloid) |
| Involvement of family members in financial planning | “Well, like most people, I just don't want to be a burden on my family, especially with kids. We're working very hard to make sure that we're debt-free and we actually have been giving the kids things, so that when the time comes, they already have things we want them to have and they don't have to go through a lot of probate and all that sort of stuff." (Patient, MCI with elevated amyloid)  "[We] notified my sister, who is the executor of the trust. And so they are aware of our health conditions and where we are. Other than that, they know there's sufficient funds. You just have to monitor to make sure that we're both mentally able to make those financial decisions” (Care partner, MCI with elevated amyloid)  “Oh, I do, my wife is, she's my rock. She really is. She's been through so much with me, physically as well as with this… She and I, she knows how I feel. She knows that there may come a time where she can't care for me, and then it's time to make a decision. My insurance is paid up, so anything ever happened to me, she's okay. She'll be okay for the rest of her life, but I just don't want to be a burden on my wife. And I do plan with her because I take her counsel more than anybody else's because as I said, we've been through a lot of things together. And even though I have children, they're separated from it and they live in other areas and they have children, I don't want them to be bothered. Really." (Patient, MCI with elevated amyloid)  “We talk to (Wife)' sister regularly. She's involved, and she gives advice. She helped take care of (Wife)' mother and (Wife)' aunt. She made a lot of the decisions for them. We talked to our oldest son, and he's much more involved. Although both sons have given me input as to help on my [inaudible], but my oldest son who is a very successful businessman is very much involved in our financial planning, and he's a trustee. If anything were to happen to me, he's trustee in our wills, in our plans, and on our investment portfolio account. He knows, and is very involved in what our personal situation is.” (Patient, MCI with elevated amyloid)  “Well, I think if it turns out there were some inexpensive custodial care that would preserve the wealth accumulated for my wife and my children and grandchildren, I might just suck it up and get into some sort of institutional setting. But those are usually pretty expensive. So I don't know. That's the real persistent looming threat. We seem to have the cognitive stuff more or less under control. There's a little slippage, but I'm still working, and it's sort of a persistent worry… So now the big threat in terms of bankruptcy is one of us having a really expensive  custodial situation.” (Patient, MCI without elevated amyloid) |
